# A Fuzzy-Ontology Based Diabetes Monitoring System Using Internet of Things

**DOI:** 10.1007/978-3-030-51517-1_25

**Published:** 2020-05-31

**Authors:** Sondes Titi, Hadda Ben Elhadj, Lamia Chaari Fourati

**Affiliations:** 8 Digital Research Centre of Sfax, Sfax, Tunisia; 9 Institut Mines-Télécom, CNRS, Paris, France; 10 Université de Sherbrooke, Sherbrooke, QC Canada; 11 Digital Research Centre of Sfax, Sfax, Tunisia; 12 University of Sfax, Sfax, Tunisia; 13 Laboratory of Technology and Smart Systems (LT2S), LR16CRNS01, Sfax, Tunisia; 14 Digital Research Center of Sfax, Sfax, Tunisia

**Keywords:** Fuzzy, Ontology, Internet of thing, Healthcare, Diabetes

## Abstract

The majority of the Internet-of-things (IoT)-based health monitoring systems adopt ontologies to represent and interoperate the huge quantity of data collected. Classical ontologies cannot appropriately treat imprecise and ambiguous knowledge. The integration of Fuzzy logic theory with ontology can effectively resolve knowledge problems with uncertainty. It considerably raises the accuracy and the precision of healthcare decisions. This paper presents a fuzzy-ontology based system using the internet of things and aims to ensure continues monitoring of diabetic patients. It mainly describes the ontology-based model and the semantic fuzzy decision-making mechanism. The system is evaluated using semantic querying. The results indicate its feasibility for effective remote continuous monitoring for diabetes.

## Introduction

The increasing number of diabetic patients place a severe burden on healthcare systems and makes their monitoring a very difficult task. According to [17], the total number of diabetic patients is expected to rise from 171 million in 2000 to 366 million in 2030. Diabetes is a group of metabolic disorders of carbohydrate metabolism characterized by the variation of blood glucose level that results from insufficient production of the hormone insulin (type 1 diabetes) or an ineffective response of cells to insulin (type 2 diabetes). It requires remote continuous monitoring to prevent emergencies and long-term complications such as cardiovascular diseases. Therefore, its treatment should focus mainly on controlling and managing blood glucose levels constantly with diet, physical exercises, and medications. New healthcare systems based on IoT offer a new effective perspective in diabetes management based on the IoT data collected. They are enable to sufficiently handle imprecise and vague information related to patient and therefore fail in describing his health condition and to recommend the appropriate drug and food, because they adopt conventional approaches such as classical ontology and fuzzy logic. The combination of ontologies and fuzzy-logic approaches can effectively resolve the problem of uncertainty of data related to diabetic patients and thus ameliorate the accuracy of system when performing decisions related to the current health condition and recommendations. This paper introduces a fuzzy-ontology-based healthcare system integrating IoT technologies. The system provides certain and precise diabetes-related decisions that allow patients to maintain a lifestyle in which the diet is coordinated with exercise and activities. The remainder of this paper is organized as follows. Section 2 presents related works. Section 3 then describes the architecture of the proposed system and the knowledge construction mechanism. Next, Sect. 4 introduces the fuzzy-ontology generating model. Section 5 describes the semantic fuzzy decision-making process for diabetes system and summarizes the evaluation results. Conclusions and perspectives are finally drawn in Sect. 6.

## Related Works

The increasing number of chronically ill aged people worldwide has drawn the attention of a diverse array of fields including Internet of Things (IoT) and Artificial Intelligence (IA), explaining why IoT based healthcare systems using ontology and fuzzy-logic have been adopted for continues real-time monitoring. For instance, Mumtaj et al. [10] proposed an IoT-based system combining ANN and fuzzy logic and aims to ensure the monitoring of elderly and supports caregivers to diagnose diseases by sending alerts in case of any abnormalities. Huang et al. [6] introduced predictive symptom checker system based fuzzy logic that helps the elderly to decisively determine the most appropriate illness and any health-related threats. Another system presented in [11] aims to evaluate the likelihood of developing heart diseases in patients. Another work presented in [3] introduced an expert fuzzy system for heart disease diagnosis. IoT allows users to share information everywhere and every time. However, the huge exploitation of the connected objects becomes a source of a mass of heterogeneous data. Ontologies play an important role to deal with the huge quantity of data by offering a semantic representation of the domain knowledge. Recently, different ontologies related to IoT based healthcare are proposed. For instance, [12] describes an ontology-based framework using the semantic IoT that provides continuous monitoring of patient status. Another work in [14] presents an IoT based system where an ontology is introduced to provide semantic interoperability among heterogeneous devices and users to ensure remote control of patient affected by chronic diseases. In [2], the authors proposed a context management system for smart environments that uses an ontology to model the uncertainty and vagueness of the contextual information collected to reach a richer inference process. Authors in [9], propose an ontology-based context management system that allows the monitoring of the elderly citizens’ behavior and the detection of risks related to mild cognitive impairments and frailty. The work proposed in [8] describes a decision support system aiming to ensure the treatment and care delivery pathways for patients with Head and Neck Cancer. In [15] a personalized ontology-based food recommendation system is proposed for supporting travelers with long-term diseases and follow a strict diet. Although classical ontologies provide a formalized and accumulated knowledge base for users to investigate and share, they cannot appropriately treat imprecise and vague knowledge for healthcare applications [18]. Thus, the combination of fuzzy logic theory with ontology is considered the solution for uncertainty. For example, the system presented in [5] enables elderly citizens suffering from chronic diseases to live safely and independently by generating more effective and accurate recommendations. [7] proposed a fuzzy expert decision system for diabetic patients. The system aims to model diabetes knowledge with uncertainty. [1, 13] propose recommender fuzzy-ontology based systems that efficiently monitor the diabetic patient and recommend appropriate foods and drugs.

## Architecture of Fuzzy Ontology-Based Healthcare System

**Fig. 1.**
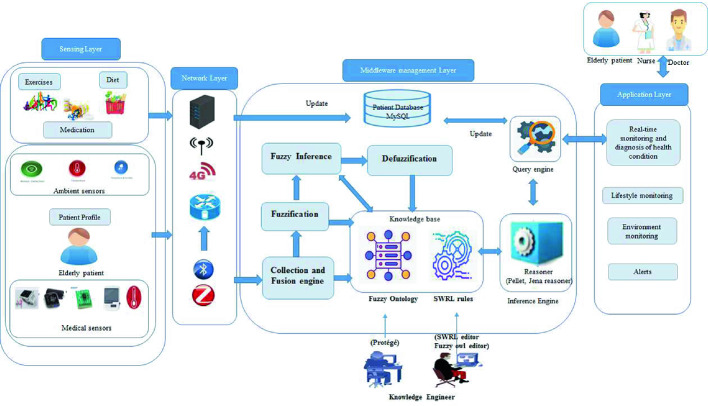
The architecture of fuzzy ontology-based healthcare system

This section describes the overall architecture of the proposed system (Fig. 1). Different medical and ambient sensors and devices are used to monitor the patient’s vital signs and his home surroundings. These measurements data combined with patient profile data, lifestyle data including diet, physical exercises and medication intake constitute the sensing layer. The data collected is transferred to the server using the interconnected IoT technologies that constitute the network layer. On the server-side, the middleware management layer, which acts as the central part of the system, is deployed. It is responsible for data collection and management, sensors and devices control and services generation. Its main Components are:

- Collection and fusion engine: receives data from medical and ambient IoT devices and other data related to patient’s profile and lifestyle (foods, medication, and exercise) and extracts features/inputs to send them to the fuzzification component and to the knowledge base.
- Database: stores patients’ profile data, symptoms, medical history, medication intake, exercises, meals and foods, examination results, etc.
- The knowledge base: comprises the fuzzy-ontology that formulates data representing the patient, his environment, his lifestyle, his examinations and rule-based reasoning model fusing SWRL and fuzzy-logic theory to deal with vagueness and uncertainty and thus ameliorates the efficiency of decisions making results.
- Fuzzification: transforms raw feature/input values to fuzzy variables. This is to determine the membership function of each variable in the set.
- Fuzzy inference: maps input variables to output variable through a number of fuzzy if-then rules.
- Defuzzification: transforms fuzzy variables to output values. These crisp values are necessary for the generation of healthcare services.
- Query engine: handles queries received from the application layer.
- Reasoning Engine: checks the consistency of raw data and deduces the high-level data from low-level data.

The results of diagnosis representing healthcare services are delivered to the end users (doctors, patient, nurses, and family members). Multiple programming interfaces are developped to display the results that answer users’ queries.

## Proposed Fuzzy-Ontology

Ontologies have been adopted in IoT-based healthcare applications, as they are capable of modeling and representing the whole concepts related to the health domain and describing the relationships among them. However, classical ontologies are unable to handle imprecise and vague knowledge and thus fail to provide accurate and efficient diagnoses. This limitation has leading us to develop a fuzzy-ontology capable of managing health-related knowledge. The ontology proposed uses fuzzy members and gives a semantic description related to diabetes disease. It supports the patient in monitoring their health condition and their lifestyle and generating efficient recommendations. It is an extension of our classic ontology proposed in [14]. Protege tool is used to develop and maintain our proposed fuzzy-ontology. It allows reasoning through different plugins: Fuzzy owl is used to add fuzzy sets to fuzzy variables. SWRL is adopted to manage fuzzy rules. DL and SPARQL queries are employed to retrieve the results and answers. The ontology includes multiple classes representing the concepts, data and object properties and fuzzy data types representing the intervals of the membership fuzzy variables. The fuzzy concepts define concepts and relations to describe uncertain and vague knowledge. The main difference between fuzzy and classic concepts is that in classic concepts the membership degree of each property is equal to 1 or 0 while in fuzzy concepts it is equal to a certain degree belongs the interval [0, 1]. The classes extended are the following:

- Fuzzy patient class: represents all the information required to supervise the condition of the diabetic elderly. It describes 5 fuzzy variables which are height, weight, gender, disease history and age. The fuzzy variable weight has the fuzzy sets “Light”, “Normal”, and “Heavy” and “Obese”. The fuzzy variable gender has the fuzzy set “Male” and “Female”. The fuzzy variable age has these sets, “Young”, “Adult” and “Old”.
- Fuzzy MedicalProperty class: describes and manages the medical observations. It has the following Fuzzy sub-classes: blood pressure, blood glucose, BMI, heart rate, temperature. These measurements are used as the input variables to identify the health condition of the patient which is the output variable. Each fuzzy variable has several fuzzy terms. For example, the blood sugar glucose variable has the fuzzy sets: (very-low 0–90, low 71–130, medium 125–154, high 142–180, very-high 165–250).
- Fuzzy Health condition class defines the patient’s health condition calculated based on medical data collected. The health condition is the output variable determined based on fuzzy input variables defining the medical measurements and the fuzzy rules. This variable has fuzzy sets “Healthy”, “Moderate” and “Serious”. The system acts automatically based on the patient’s health condition: If it is healthy, the system indicates to the patient to maintain his lifestyle. If it is moderate, the system recommends the appropriate drugs, foods and physical exercises required for the patient to establish his normal health condition and notify the corresponding caregiver to do the regular health services. If it is serious, the system generates alarms to call the medical staff and recommends different foods and drugs.
- Fuzzy Food class: defines the food eaten by a diabetic patient. Foods are distributed in meals. According to the nutritionists, a diabetic patient should maintain a healthy diet that allows him to maintain a normal blood glucose level. The meal eaten is considered healthy or UnHealthy based on the percentage of carbohydrate PC, protein PP, and fat consumed PF, BMI, the difference between the calories consumed by the patient and the planned total calories required for patient’s body defined by nutritionists DCP. The total calories needed to maintain or lose weight is calculated based on the basal metabolic rate BMR and the activity level. The BMR is calculated based on patient’ age, gender, weight and height using Mifflin St Jeor formula [4]. The nutritionists recommend that the planned total calories should be divided into the five meals: Breakfast, breakfast, snack 1, lunch, snack 2, and dinner with the respective percentage: 25%, 12.5%, 25%, 12.5%, and 25%. For each meal, the number of calories should be distributed in three nutrients, which are carbohydrates, fat, and protein with the respective percentage 50%, 30%, and 20%. Therefore, four fuzzy variables are described by this class which are PC, PP, PF, DPC. These variables are considered as inputs variables combined with the fuzzy variables Age, BMI and physical activity to deduce if the diet is healthy or not which is expressed by the fuzzy variable Diet status.
- Fuzzy physical activity: expresses the level of physical activity practiced by the diabetic patient. The level of physical activity is used to calculate the total calories needed by the diabetic patient to maintain or lose weight. In fact, according to the level of activity, a factor is multiplied by the BMR as following: little = BMR * 1.2, light = BMR * 1.375, moderate = BMR * 1.55, strenuous = BMR * 1.725, extra strenuous = BMR * 1.9.

## Health Condition and Diet Status Calculation Process

This section details the process calculation of health condition and diet status values for the diabetic patient using the following components: fuzzy-ontology, fuzzification, fuzzy rule-base, fuzzy inference, and defuzzification. The crisp inputs related to patient’s profile and sensors data are collected and then fuzzified using the fuzzy sets of each variable. Health condition is calculated using blood glucose, blood pressure, BMI, heart rate, body temperature fuzzy inputs. Diet Status is calculated based on PC, PP, PF, DCP, activity, Age, BMI. The fuzzification step is proceeded by the fuzzy inference step. This later uses the fuzzy-ontology and a fuzzy-rule base that includes a set of fuzzy-rules. The proposed fuzzy rules are categorized in: (1) Rules to determine the health condition of the patient (2) Fuzzy rules to determine the status of diet consumed by the patient. (3) Rules to determine the BMR and planned calories needed by the patient according to his physical activity level, height, weight, and age. (4) Rules to recommend drugs, foods and physical activity according to health and diet conditions calculated. Following, are example of two SWRL rules. Rule 1 deduces the health status of the patient based on his physiological signs and generates Health services. Rule 2 deduces the diet status based on the composition of meals eaten by the patient, his activity and the difference between the planned and consumed calories, and generates appropriate recommendations. The rules determining the status of diet are implemented according to these two conditions: (1) the diet is more healthy if PC, PF, PP, activity are more balanced and (2) the planned caloric intake is closer to the one consumed. The Fuzzy inference is based on Mamdani’s method to determine the fuzzy output variable and send it to the defuzzyfier. The defuzzyfier adopts ‘Center of gravity’ [16] method to deduce the crip value of the output variable.

**Rule 1:** Patient(?p), HasBloodGlucose(?p, HighBG), HasBloodPressure(?p, HighBP), HasHeartBeat(?p, HighHB), HasBMI(?p, OverweightBMI), HasBodyTemperature(?p, NormalBT), greaterThan(?HighBG, 180), SystolicBPValue(?HighBP, ?s), DiastolicBPValue(?HighBP,?d), greaterThan(?d, 86), lessThan(?d, 90), greaterThan(?s, 131), lessThan(?s, 139), greaterThan(?NormalBT, 37), lessThan(?NormalBT, 38), greaterThan(?HighHB, 100), greaterThan(?OverweightBMI, 38), lessThan(?OverweightBMI, 40), Alarm(?a), EmergencyButton(?e), UseActuatingDevice(?p, ?e) -> HasHealthCondition(?p, Serious), HasAlarm(?p, ?a), HasServiceMessage(?a, “You are in danger, Call a doctor”), HasActuatorState(?e, “Device is swiched On”)

**Rule 2:** Patient(?p), HasAge(?p, Old), HasBMI(?p, OverWeight), HasPC(?p, HighPC), HasPP(?p, HighPP), HasPF(?p, LowPF), HasDCP(?p, MLUnAcceptableCDP), greaterThan(?Old, 65), HasActivity(?P, LightAC), Recommandation(?R), greaterThan(?OverWeight, 38), lessThan(?OverWeight, 40), greaterThan(?HighPC, 65), lessThan(?HighPC, 100), greaterThan(?HighPP, 20), lessThan(?HighPP, 100), lessThan(?LowPF, 25), greaterThan(?LightAC, 8), lessThan(?LightAC, 30), greaterThan(?MLUnAcceptableCDP, 50), lessThan(?MLUnAcceptableCDP, 200)-> HasDietStatus(?p, UnHealthy), HasRecommandation(?p, ?R), HasServiceMessage(?R, “Do more exercises, Eat More Fat, Less Carbohydrate, Less Protein”), NeedFood(?p, LowCalorie).

The proposed system is implemented on java. Fuzzy components are implemented using jfuzzylogic plugin to calculate the output variables, which are health condition and diet status. The ontology is evaluated based on the querying-answering approach using DL and SPARQL queries and is then integrated into the java application using Jena API. The evaluation includes: (1) Technical evaluation to check the consistency and the coherence of the ontology using Jena reasoner as well as the response time that the system takes to execute user queries and display the result. Results show that the time consumed depends on the number of inputs that the query needs to calculate the output. The more the inputs are, the more the response time is. (2) Functional evaluation, which evaluates the efficiency of the system and the accuracy level of the decisions for which the system was queried. Results demonstrate that the accuracy of our system can reach 100% for diet status related queries, 96% for health condition related queries and on average 94% for queries related recommandations. Therefore our system is capable of acting more similar to human expertise.

## Conclusion

This paper proposes a fuzzy-ontology based diabetic monitoring system using IoT technology. The fuzzy-logic is adopted to infer the health condition and the diet status for the patient and then presents the result to the ontology to generate convenient recommendations. Evaluation indicates that the performance of the system is increased considerably and the system gives results with more accuracy compared to the system using classic ontology.
